# Sex estimation from skull measurements of a contemporary Japanese population using three-dimensional computed tomography images

**DOI:** 10.1007/s00414-024-03319-8

**Published:** 2024-08-30

**Authors:** Yumi Hoshioka, Suguru Torimitsu, Yohsuke Makino, Daisuke Yajima, Fumiko Chiba, Rutsuko Yamaguchi, Go Inokuchi, Ayumi Motomura, Shigeki Tsuneya, Hirotaro Iwase

**Affiliations:** 1 Department of Legal Medicine, Graduate School of Medicine, Chiba University, 1-8-1 Inohana, Chuo-ku, Chiba, 260-8670 Japan; 2 Department of Forensic Medicine, Graduate School of Medicine, The University of Tokyo, 7-3-1 Hongo, Bunkyo-ku, Tokyo, 113-0033 Japan; 3 Department of Forensic Medicine, School of Medicine, International University of Health and Welfare, 4-3 Kozunomori, Narita, 286-8686 Japan; 4 Department of Legal Medicine, Nihon University School of Medicine, 30-1 Oyaguchi Kami-cho, Itabasi-ku, Tokyo, 173-8610 Japan

**Keywords:** Forensic anthropology population data, Multidetector computed tomography, Skull, Japanese, Sex estimation

## Abstract

In this study, we assessed the sexual dimorphism of the contemporary Japanese skull and established sex discriminant function equations based on cranial measurements using three-dimensional (3D) computed tomography (CT) images. The CT images of 263 corpses (142 males, 121 females) that underwent postmortem CT scanning and subsequent forensic autopsy were evaluated. Twenty-one cranial measurements were obtained from 3D CT reconstructed images, which extracted only bone data. We performed descriptive statistics and discriminant function analyses for the measurements. Nineteen measurements were significantly larger in males, suggesting sexual dimorphism of the Japanese skulls. Univariate discriminant function analyses using these measurements showed a sex classification accuracy of 57.8–88.2%, and bizygomatic breadth provided the highest correct prediction rate. Multivariate discriminant function analyses offered the most accurate model using seven variables with an estimation rate of 93.9%. Our results suggest that cranial measurements based on 3D CT images may help in the sex estimation of unidentified bodies in a contemporary Japanese population.

**Supplementary Information:**

The online version contains supplementary material available at 10.1007/s00414-024-03319-8.

## Introduction

Unidentified individuals who are incomplete, decomposed, or skeletonized are often encountered during forensic investigations. When anthropologists investigate such corpses, determining biological profiles, such as the sex, age, stature, and ancestry, from skeletal elements is critical [1]. Notably, accurate sex estimation is essential for identifying an unknown body because other profiles are usually estimated using sex-specific standards [2, 3].

The pelvis is well known as the most reliable bone for estimating sex [4, 5]; however, other skeletal regions should be useful when the pelvis is lost or damaged. The skull has been reported to be relatively well-preserved even after centuries [6, 7]. Evaluating the skull size and robusticity has been a traditional and easy method for sex determination [5, 8, 9]. However, such qualitative analyses tend to be subjective and can lead to large interobserver errors [10]. Conversely, metric analyses for cranial sex estimation can provide more objective results [3, 11]. The craniometric sex determination was introduced in the middle of the twentieth century. Giles and Elliot [12] used the metric approach and showed sexual dimorphism of the skull and multivariate discriminant functions with high classification accuracy. Subsequently, discriminant function analyses have been performed using cranial measurements in different populations, and these publications revealed a practical sex estimation with an accuracy of over 80% [11, 13–15].

Previous studies have shown that a higher sex classification accuracy is possible when population-specific discriminant function equations are used [16–18], probably because genetic and environmental factors influence the bone growth and development [19]. Secular changes in bones are also discussed, and poor classification results have been reported when these measurements were applied to discriminant function formulae made from morphometric analyses in a different period [11, 16]. However, few studies on sex-determining methods use contemporary Japanese cranial measurements [20, 21]. Işcan et al. [20] performed discriminant function analyses using measurements of Japanese skulls macerated between 1960 and 1970 and showed a prediction accuracy of 83.7% using seven measurements.

Postmortem computed tomography (PMCT) using a multidetector CT scanner has been recently performed in some forensic institutes [22]. Researchers can immediately create three-dimensional (3D) images using the CT values and obtain bone morphological information and measurements. In addition, previous studies have revealed higher morphometric sex classification accuracy using CT images than traditional equipment like calipers [23, 24]. These advantages of using CT images have led to more anthropological studies of sex estimation by bone measurements using 3D CT images of the modern population [3, 18, 25–27]. However, no study has been conducted on sex determination using cranial measurements based on 3D CT images of the modern Japanese population.

Therefore, in this study, we aimed to investigate cranial dimorphism and develop craniometric standards for sex estimation in a contemporary Japanese population obtained using 3D CT images.

## Materials and methods

The study protocol was approved by the Ethics Committees of Chiba University (No. 2819) and the University of Tokyo (No. 10835). The requirement for approval from the subjects’ relatives was waived.

PMCT scanning and subsequent forensic autopsy were performed on 263 Japanese corpses of known age and sex (142 males, mean age = 53.0 ± 15.0 years; 121 females, mean age = 54.7 ± 17.8 years) at the Education and Research Center of Legal Medicine (Department of Legal Medicine) at Chiba University between October 2011 and September 2016 and at the Department of Forensic Medicine at the University of Tokyo between September 2016 and December 2017. Exclusion criteria were skull fractures, neck injuries, burn injuries, and acquired or congenital abnormalities.

PMCT scanning was performed with a 16-row detector CT system (Eclos; FUJIFILM Healthcare Systems Corporation, Tokyo, Japan). The scanning protocol was as follows: collimation of 0.625 mm, reconstruction interval of 0.625 mm, tube voltage of 120 kV, tube current of 200 mA, and rotation time of 1/s. A hard filter was used for image reconstruction. Image data were processed on a workstation (Synapse Vincent; Fujifilm Medical, Tokyo, Japan) to obtain orthogonal multiplanar reconstruction and volume-rendered images.

**Fig. 1:**
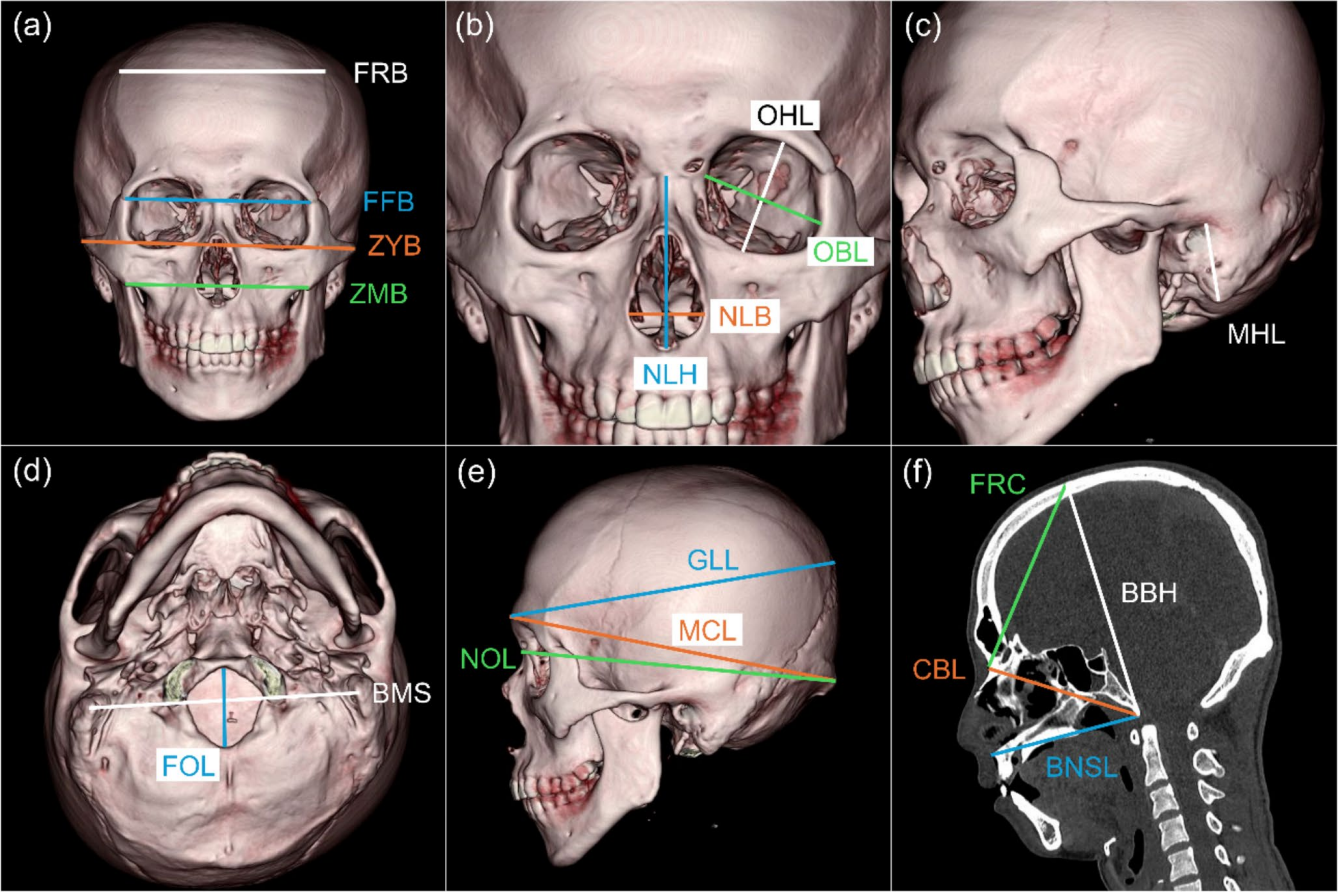
Reconstructed three-dimensional computed tomography (3D CT) images (**a**–**e**) and a sagittal plane image (**f**) of the skull showing measurements (see Online Resource 2 for definition); (**a**) from the anterior side; (**b**) expanded anterior side; (**c**) from the anterior left side; (**d**) from the inferior side; (**e**) from the left side. OHL, OBL (**b**), and MHL (**c**) were measured for each side. CBL, BBH, BNSL, and FRC (**f**) were actually measured using 3D CT images

A 3D CT reconstructed image that extracted only bone data based on the CT value data was used for assessment. Based on previous studies [18, 28–30], 21 measurements were performed on each skull using electronic cursors to the nearest 0.1 mm (Online Resources 1, 2, and Fig. 1). The measurements were acquired on the 3D rendered model alone. A subset of 20 subjects was randomly selected; the author re-collected subset data during two different sessions separated by a 2-week interval to assess intraobserver error, and a co-author collected the subset data to assess interobserver error. Subsequently, the technical error of measurements, relative technical error of measurements (rTEM, %), and coefficient of reliability (R) were calculated. The acceptance ranges of rTEM using beginner anthropometrist levels for intraobserver and interobserver errors were < 1.5% and < 2.0%, respectively [31]. An R value demonstrates the proportion of the between-subject variance free of measurement errors; an R value of > 0.95 was considered sufficiently precise [32, 33].

All statistical analyses were performed using SPSS version 21.0 computer software (IBM, Armonk, NY, USA) and JMP^®^ 15 (SAS Institute Inc., Cary, NC, USA). Means, standard deviations, and ranges were calculated for age, stature, and all cranial measurements. The Anderson–Darling test was used to determine whether each cranial measurement was normally distributed across subjects. If a set of cranial measurements was normally distributed, Welch’s t-test was used to compare the mean differences between the sexes in these variables. However, if a set of cranial measurements was not normally distributed, the Mann–Whitney U test was used to determine whether there was a difference between the sexes in these variables. Statistical significance was set at a *p*-value of < 0.05.

Univariate discriminant function equations for sex classification were created for each cranial measurement. In addition, stepwise discriminant function equations were derived for all cranial measurements to provide the most accurate sex classification. Variable selection was performed based on F values (3.84 to enter and 2.71 to remove). The sectioning points were calculated by adding two group centroids and dividing the total by two. In addition, Wilk’s lambda was calculated to show how well each independent variable level separated subjects into males and females; the scale ranged from 0 to 1, where 0 represents total discrimination, and 1 represents no discrimination. The accurate classification rates of the derived discriminant function equations were calculated using the leave-one-out cross-validation procedure.

## Results

Table 1 presents the rTEMs and R values of the 21 cranial measurements for assessing intraobserver and interobserver errors. For intraobserver errors, the rTEMs and R values of 19 measurements were < 1.5% (0.226–1.250%) and > 0.95 (0.952–0.995), respectively, whereas the rTEMs of NLB (1.501%) and RMHL (1.687%), and R values of ROHL (0.918) and NLB (0.921), were slightly out of the acceptable ranges. For interobserver errors, rTEMs of all measurements were < 2.0% (0.203–1.190%), and R values were > 0.95 (0.952–0.994) except for LOBL (0.947) and ROBL (0.940).

**Table 1:** The relative technical error of measurement (rTEM) and coefficient of reliability (*R*) (*n* = 20)

Variable	Intraobserver			Interobserver	
	rTEM	*R*		rTEM	*R*
FRB	0.507%	0.988		0.455%	0.963
ZYB	0.470%	0.979		0.323%	0.969
LOHL	0.933%	0.972		0.885%	0.957
ROHL	1.140%	0.918		0.901%	0.954
LOBL	0.913%	0.967		0.842%	0.947
ROBL	1.097%	0.983		0.886%	0.940
ZMB	0.777%	0.970		0.565%	0.955
FFB	0.557%	0.980		0.469%	0.952
NLB	1.501%	0.921		1.170%	0.954
LMHL	1.250%	0.973		1.126%	0.952
RMHL	1.687%	0.973		1.190%	0.952
BMS	0.494%	0.988		0.385%	0.962
MCL	0.389%	0.987		0.221%	0.983
GLL	0.407%	0.984		0.237%	0.977
CBL	0.338%	0.985		0.301%	0.990
BBH	0.397%	0.993		0.270%	0.976
BNSL	0.420%	0.981		0.306%	0.987
FOL	0.880%	0.971		0.665%	0.976
FRC	0.455%	0.988		0.316%	0.969
NOL	0.361%	0.952		0.203%	0.984
NLH	0.226%	0.995		0.456%	0.994

Table 2 shows the descriptive statistics of the mean, standard deviation, and the range of age, stature, and 21 measurements. The mean values of stature and 19 measurements were significantly larger in males (*p* < 0.05). There were no significant differences in the mean LOHL and ROHL between the sexes; therefore, they were excluded from the subsequent sex prediction analyses.

**Table 2:** Descriptive statistics and results of comparison using *t*-tests for age, stature, and 21 measurements

Variable	Male (*n* = 142)			Female (*n* = 121)			*p*-value
	Mean ± SD	Range		Mean ± SD	Range		
Age (years)	53.0 ± 15.0	21–87		54.7 ± 17.8	21–89		0.411
Stature (cm)	170.3 ± 5.7	155.0–182.0		156.5 ± 6.0	142.0–171.0		< 0.001*
FRB (mm)	116.5 ± 8.2	85.9–132.5		113.6 ± 7.2	87.6–129.6		0.003*
ZYB (mm)	139.8 ± 4.8	124.4–152.3		130.3 ± 4.2	122.5–144.2		< 0.001*
LOHL (mm)	36.2 ± 2.0	31.7–41.1		36.0 ± 2.0	30.1–41.1		0.360
ROHL (mm)	36.1 ± 2.0	31.3–40.9		35.9 ± 2.1	29.4–41.1		0.498
LOBL (mm)	41.3 ± 2.1	36.0–48.9		39.6 ± 2.2	35.1–45.2		< 0.001*
ROBL (mm)	41.6 ± 2.4	35.8–50.5		39.8 ± 2.4	35.6–46.0		< 0.001*
ZMB (mm)	101.6 ± 5.7	88.5–121.7		94.8 ± 4.6	83.7–111.3		< 0.001*
FFB (mm)	99.4 ± 3.5	90.4–108.6		94.5 ± 3.6	86.3–105.9		< 0.001*
NLB (mm)	26.9 ± 2.1	21.8–33.0		25.7 ± 2.0	21.3–31.0		< 0.001*
LMHL (mm)	34.8 ± 3.3	27.6–44.0		30.0 ± 2.8	23.4–37.2		< 0.001*
RMHL (mm)	34.9 ± 3.3	26.5–41.9		29.9 ± 2.9	22.2–35.7		< 0.001*
BMS (mm)	109.7 ± 5.8	96.9–129.0		103.4 ± 5.3	83.2–122.0		< 0.001*
MCL (mm)	181.9 ± 6.1	165.4–197.8		170.4 ± 6.5	149.4–192.2		< 0.001*
GLL (mm)	178.4 ± 6.1	163.9–194.8		167.6 ± 6.3	148.8–184.1		< 0.001*
CBL (mm)	104.7 ± 4.5	92.8–121.7		96.8 ± 4.3	86.3–108.5		< 0.001*
BBH (mm)	143.7 ± 5.1	128.5–157.6		136.8 ± 5.2	121.2–151.1		< 0.001*
BNSL (mm)	94.5 ± 5.0	80.7–111.4		87.5 ± 4.5	75.2–103.3		< 0.001*
FOL (mm)	38.9 ± 2.7	31.5–45.3		36.7 ± 2.4	31.5–43.9		< 0.001*
FRC (mm)	115.5 ± 4.5	105.5–124.9		109.9 ± 4.6	100.6–121.5		< 0.001*
NOL (mm)	179.0 ± 5.7	161.6–193.3		169.0 ± 6.2	149.9–188.0		< 0.001*
NLH (mm)	56.4 ± 3.2	48.3–67.8		51.9 ± 3.7	43.0–72.1		< 0.001*

* Significant

Table 3 presents the results of the univariate and stepwise discriminant function analyses of the cranial measurements. If the discriminant function score is larger than the sectioning point, the subject is assigned as male; otherwise, it is assigned as female. The correct prediction rate of the univariate discriminant function analysis was highest for ZYB (88.2%), followed by CBL (84.0%). Accuracy rates of approximately 80% were obtained with the other six variables (MCL, LMHL, BNLS, NOL, FFB, and GLL). Stepwise analysis using seven measurements (ZYB, RMHL, MCL, CBL, FOL, NOL, and NHL) showed a more accurate estimation rate (93.9%) and a lower Wilks’ lambda than those of the univariate analyses.

**Table 3:** Univariate and stepwise discriminant function analyses

Variable	Unstandardized coefficient	Fisher’s linear DF^a^		Group centroid			Sectioning point	Wilk’s lambda		Correct prediction rates (%)					Posterior probability (%)
		Male	Female		Male	Female				Male	Female	Overall	Sex bias		Overall
Univariate															
FRB	0.129	1.929	1.881		0.171	−0.200	−0.015	0.967		59.9	55.4	57.8	4.5		58.6
Constant	−14.818	−113.033	−107.536												
ZYB	0.222	6.889	6.417		0.979	−1.149	−0.085	0.469		88.0	88.4	88.2	−0.4		88.2
Constant	−30.058	−482.350	−418.565												
LOBL	0.472	9.196	8.811		0.376	−0.441	−0.033	0.857		66.9	62.8	65.0	4.1		64.0
Constant	−19.119	−190.712	−175.120												
ROBL	0.418	7.272	6.952		0.351	−0.412	−0.031	0.873		66.2	68.6	67.3	−2.4		66.3
Constant	−17.047	−152.037	−139.045												
ZMB	0.190	3.684	3.438		0.596	−0.700	−0.052	0.704		71.1	76.9	73.8	−5.8		73.8
Constant	−18.751	−187.858	−163.619												
FFB	0.282	7.908	7.516		0.639	−0.750	−0.056	0.675		77.5	81.0	79.1	−3.5		79.1
Constant	−27.394	−393.616	−355.666												
NLB	0.497	6.627	6.343		0.263	−0.309	−0.023	0.924		59.9	57.9	58.9	2.0		58.9
Constant	−13.079	−89.704	−82.234												
LMHL	0.326	3.696	3.182		0.725	−0.850	−0.063	0.617		82.4	78.5	80.6	3.9		80.6
Constant	−10.616	−65.000	−48.379												
RMHL	0.322	3.608	3.093		0.737	−0.865	−0.064	0.609		73.9	79.3	76.4	−5.4		76.8
Constant	−10.482	−63.625	−46.938												
BMS	0.180	3.566	3.362		0.521	−0.611	−0.045	0.757		72.5	75.2	73.8	−2.7		72.2
Constant	−19.255	−196.223	−174.484												
MCL	0.159	4.615	4.324		0.841	−0.986	−0.073	0.545		83.1	81.8	82.5	1.3		82.1
Constant	−28.131	−420.356	−369.098												
GLL	0.161	4.649	4.367		0.804	−0.944	−0.070	0.566		79.6	78.5	79.1	1.1		79.5
Constant	−27.996	−415.412	−366.584												
CBL	0.227	5.381	4.976		0.822	−0.964	−0.071	0.556		84.5	83.5	84.0	1.0		84.4
Constant	−22.909	−282.271	−241.481												
BBH	0.193	5.350	5.093		0.615	−0.721	−0.053	0.691		78.9	74.4	76.8	4.5		76.8
Constant	−27.112	−385.085	-348.931												
BNSL	0.210	4.180	3.870		0.678	−0.795	−0.059	0.648		78.9	80.2	79.5	−1.3		81.0
Constant	−19.195	−198.148	−169.958												
FOL	0.385	5.772	5.445		0.390	−0.457	−0.034	0.848		68.3	67.8	68.1	0.5		68.1
Constant	−14.585	−112.807	−100.481												
FRC	0.221	5.620	5.345		0.572	−0.672	−0.050	0.721		70.4	73.6	71.9	−3.2		72.2
Constant	−24.903	−325.184	−294.271												
NOL	0.169	5.107	4.823		0.773	−0.908	−0.068	0.586		81.0	77.7	79.5	3.3		80.2
Constant	−29.459	−457.680	−408.275												
NLH	0.293	4.850	4.468		0.598	−0.702	−0.052	0.702		73.2	76.9	74.9	−3.7		74.9
Constant	−15.937	−137.407	−116.743												
Stepwise analysis															
ZYB	0.095	5.070	4.776		1.431	−1.680	−0.125	0.292		93.0	95.0	93.9	−2.0		94.3
RMHL	0.133	2.264	1.850												
MCL	0.187	−1.969	−2.552												
CBL	0.051	1.161	1.003												
FOL	0.079	2.628	2.384												
NOL	−0.147	5.683	6.142												
NLH	0.065	1.823	1.622												
Constant	−36.137	−887.406	−775.375												

^a^ Discriminant function

## Discussion

In this study, the rTEMs and R values for intraobserver and interobserver errors were within acceptable ranges for most measurements. Franklin et al. [34] suggested that any measurement with an R value < 0.90 with the associated rTEM > 5% should be treated cautiously; however, we had no measurement in these ranges. In addition, the intraobserver and interobserver errors were low in our previous study that evaluated the correlation between stature and skull measurements using the same CT scanning protocol [35]. Considering these results, cranial measurements using 3D CT images may have no significant technical errors and are highly reproducible.

The values of 19 of the 21 cranial measurements in men were significantly higher than those in women, indicating that the skull is strongly dimorphic in a contemporary Japanese population, and the craniometric method could be used for sex estimation in a modern Japanese population. These measurements have been similarly reported to be sexually dimorphic in different populations [3, 11, 13–15, 18, 25, 36–38]. Previous studies have shown that orbital height had no significant difference between the sexes [14, 18, 36], and these results are consistent with those of our study.

ZYB showed the highest correct prediction rate of 88.2% in the univariate discriminant function analyses. Notably, several previous studies in various populations have also reported that ZYB is the most accurate cranial measurement for sex estimation (79.4–85.0%) [3, 11, 14, 18, 38]. CBL, MCL, and LMHL showed relatively high prediction rates of > 80% (80.6–84.0%) in this study, which are highly dimorphic next to ZYB in previous studies [3, 11, 14, 18]. Therefore, these accurate classification rates in this study were comparable to or somewhat higher than those in previous studies.

Previous studies have included these dimorphic measurements in multivariate discriminant function equations, and the classification accuracy has been demonstrated to be higher than that of single-variable models. Approximately 85.7–90.3% sexing accuracy was obtained from multivariate prediction formulas using two to eleven measurement variables in various populations [3, 11, 13–15, 18, 25, 36–38]. The present study’s stepwise analysis using seven variables, including ZYB, MCL, and CBL, showed the highest prediction rate of 93.9%, indicating that the equation may be useful for sex estimation. Notably, various variables and statistical methods were used; however, our correct prediction rate was higher than that in previous reports. It is also higher than the previous results of Japanese sex estimation using measurements of skulls with traditional equipment [20, 21]. Our results indicate that accurate sex determination may be achieved when the complete skull is available for forensic analysis.

All subjects in our study were aged > 16 years, and separate analyses for different age groups were not conducted. However, in the somatometric stature estimation study of Japanese skulls, Chiba and Terazawa [39] revealed that the correlation coefficient of the regression equation for subjects aged < 70 years was higher than that for all subjects, indicating that the morphological structure of the skull may change with age. In another report for sex estimation using skulls, Gillet et al. [36] analyzed the subjects separately for ages > 40 and ≤ 40. They showed that the derived multivariate models for the highest accuracy in each age group differed, indicating the possibility that bone resorption due to changes in estrogen and other hormone levels was involved. Suppose the classification accuracy derived from a discriminant function formula for a specific age group is higher than that for the whole age group; the age-group specific function formula might be preferred when the unidentified body’s age can be roughly estimated using other bone features or cranial sutures [40–42]. Therefore, further studies on sex estimation using Japanese skulls according to age are required.

Secular changes in bones have been observed in previous reports from different populations, and various factors, including environment, nutrition, socioeconomic status, and heterosis, have been thought to be responsible for the cranial form [15, 43]. However, the influence of these possible factors on sexual dimorphism has not been quantified [37], and the trends and extents of secular changes vary among populations [15]. Kouchi [43] reported that brachycephalization (the increases in head height and breadth) was observed in Japanese and might have been accelerated by improved nutrition in prenatal and early postnatal life. Therefore, continuous updates of the discriminant function equations for sex estimation might be required in every population.

Notably, some previous studies used sliding or spreading calipers for dry skull measurements in sex determination [11, 15]. However, in this study, metric data of the skull were obtained from PMCT images. Stull et al. [44] performed cranial measurements with three approaches on the same bones: CT images with soft tissues before autopsy, CT images of the dry bones after soft tissue removal, and caliper measurements of dry bones. They revealed that the differences in measured values between each procedure were generally within 2 mm, which was an acceptable amount of error for anthropologists. In addition, Djorojevic et al. [45] conducted validation tests and showed no significant differences in sex prediction accuracy between the functions acquired from 3D CT images and those obtained from dry bones. These results indicate that skeletal measurements using 3D CT images of corpses are comparable to measurements acquired on dry bones.

CT scanning for morphometric studies also eliminates the time-consuming and destructive maceration procedures [25], requires a small physical space to store the data permanently, and allows researchers to perform repeated measurements, resulting in high reproducibility. Furthermore, CT image measurements can be performed anywhere in the world using Digital Imaging and Communication in Medicine [44]. Therefore, PMCT may be useful for acquiring and applying contemporary skeletal information to forensic anthropology.

This study has certain limitations. First, detailed factors, such as nutrition and living conditions, that might influence cranial growth were not considered. Second, measurements were conducted manually, which may cause lower measurement accuracy depending on the researchers’ experience and the software used. A previous study showed that a semi-automatic bone measuring method using a specific software with artificial intelligence successfully measured 3D CT reconstructed femur images with low intraobserver and interobserver errors and shorter measurement time [46]. Further development of automatic measuring software for bone images, including skulls, is required.

## Conclusion

Our study showed that cranial measurements obtained from 3D CT images in a contemporary Japanese population revealed sexual dimorphism of the skull and allowed us to acquire sex discriminant function equations with high accuracy. Multivariate discriminant function analyses using seven measurements showed the highest classification accuracy of 93.9%. However, similar investigations of sex estimation using cranial CT images should be conducted in other populations to validate these results.

## Electronic supplementary material

Below is the link to the electronic supplementary material.

Supplementary Material 1

Supplementary Material 2

## Data Availability

Not applicable.
