# Supplementary material for: Sex estimation from skull measurements of a contemporary Japanese population using three-dimensional computed tomography images
Source: Int J Legal Med. 2024 Aug 30;139(1):383–91. doi: 10.1007/s00414-024-03319-8 (PMC11732883; doi:10.1007/s00414-024-03319-8)
Supplement: Supplementary file 1 — Supplementary Material 1 [file 414_2024_3319_MOESM1_ESM.pdf]

Electronic supplementary material 1

Article title: Sex estimation from skull measurements of a contemporary Japanese population using three-dimensional computed tomography images

Journal: International Journal of Legal Medicine

Authors: Yumi Hoshioka[a], Suguru Torimitsu[ab], Yohsuke Makino[ab], Daisuke Yajima[ac], Fumiko Chiba[ab], Rutsuko Yamaguchi[bd], Go Inokuchi[ab], Ayumi Motomura[abc], Shigeki Tsuneya[ab], Hirotaro Iwase[a]

[a] Department of Legal Medicine, Graduate School of Medicine, Chiba University

[b] Department of Forensic Medicine, Graduate School of Medicine, The University of Tokyo

[c] Department of Forensic Medicine, School of Medicine, International University of Health and Welfare

[d] Department of Legal Medicine, Nihon University School of Medicine

Corresponding author:

Yumi Hoshioka

Department of Legal Medicine, Graduate School of Medicine, Chiba University

Email address: yhoshioka@chiba-u.jp

Definitions of the skull landmarks

Landmark	Definition
Bilateral landmarks	
Frontoparietaltemporale (fpt)	Frontoparietal suture at the superior temporal line intersection
Zygion (zy)	Instrumentally determined as the most lateral point on the zygomatic arch
Superior orbital margin (os)	The point on the superior orbital margin that meets with the orbit's short axis, perpendicular to the superior orbital margin
Inferior orbital margin (oi)	The point on the inferior orbital margin that meets with the orbit's short axis, perpendicular to the superior orbital margin.
Dacryon (d)	The point on the medial border of the orbit that marks the suture junction between the frontal, maxillary, and lachrymal bones
Ectoconchion (ec)	The intersection of the most anterior surface of the orbit's lateral border and a line bisecting the orbit along its long axis (parallel to the superior margin)

Zygomaxillare (zm)	The most anterior point on the zygomaticomaxillary suture found on the bone's lateral and inferior border. The intersection of the suture and the limit of the attachment to the masseter muscle.
Fronto-zygomaticorbitale (fo)	The point where the frontozygomatic suture crosses the lateral orbital margin
Alare (al)	The most lateral point on the nasal aperture in a transverse plane
Porion (po)	The highest point on the superior margin of the external auditory meatus
Mastoidale (ms)	The most inferior point on the mastoid process
Midline landmarks	
Glabella (g)	The most forward-projecting point of the forehead in the midline at the level of the supraorbital ridges and above the nasofrontal suture
Opisthocranion (op)	Instrumentally determined most posterior point of the skull not on the external occipital protuberance
Lambda (l)	The point where the sagittal and lambdoid sutures intersect
Basion (ba)	The midline point on the anterior margin of the foramen magnum
Nasion (n)	The junction of the internasal suture with the nasofrontal suture in the median plane
Bregma (b)	The point of intersection between the coronal and sagittal suture at the midline
Nasospinale (ns)	The point where a line drawn between the inferiormost points of the nasal aperture crosses the midsagittal line
Opisthion (o)	The midline point at the posterior margin of the foramen magnum
