# Supplementary material for: Sex estimation from skull measurements of a contemporary Japanese population using three-dimensional computed tomography images
Source: Int J Legal Med. 2024 Aug 30;139(1):383–91. doi: 10.1007/s00414-024-03319-8 (PMC11732883; doi:10.1007/s00414-024-03319-8)
Supplement: Supplementary file 2 — Supplementary Material 2 [file 414_2024_3319_MOESM2_ESM.pdf]

Electronic supplementary material 2

Article title: Sex estimation from skull measurements of a contemporary Japanese population using three-dimensional computed tomography images
Journal: International Journal of Legal Medicine

Authors: Yumi Hoshioka[a], Suguru Torimitsu[ab], Yohsuke Makino[ab], Daisuke Yajima[ac], Fumiko Chiba[ab], Rutsuko Yamaguchi[bd], Go Inokuchi[ab], Ayumi Motomura[abc], Shigeki Tsuneya[ab], Hirotaro Iwase[a]

[a] Department of Legal Medicine, Graduate School of Medicine, Chiba University
[b] Department of Forensic Medicine, Graduate School of Medicine, The University of Tokyo
[c] Department of Forensic Medicine, School of Medicine, International University of Health and Welfare
[d] Department of Legal Medicine, Nihon University School of Medicine

Corresponding author:
Yumi Hoshioka
Department of Legal Medicine, Graduate School of Medicine, Chiba University
Email address: yhoshioka@chiba-u.jp

Definitions of the measurements in the present study

Measurement	Landmarks	Definition
Frontal breadth (FRB)	fpt-fpt	The breadth at the coronal suture, perpendicular to the median plane, at the temporal line
Bizygomatic breadth (ZYB)	zy-zy	The maximum breadth across the zygomatic arches perpendicular to the median plane
Left and right orbit height (LOHL and ROHL)	os-oi	The height between the orbit's upper and lower borders, perpendicular to the orbit's long axis and bisecting it
Left and right orbit breadth (LOBL and ROBL)	d-ec	Breadth from the ectoconchion to dacryon, approximating the longitudinal axis that bisects the orbit into equal upper and lower parts
Bimaxillary breadth (ZMB)	zm-zm	The breadth across the maxillae between the zygomaxillare

Bifrontal breadth (FFB)	fo-fo	The breadth across the face between the frontozygomaticorbitale on each side, the most anterior point on the frontomalar suture
Nasal breadth (NLB)	al-al	The distance between the anterior edges of the nasal aperture at its widest extent
Left and right mastoid height (LMHL and RMHL)	po-ms	The length of the mastoid process below, and perpendicular to, the eye-ear plane in the vertical plane
Bimastoidale (BMS)	ms-ms	Direct distance between the two mastoidale
Maximum cranial length (MCL)	g-op	Distance between the glabella and opisthocranion in the midsagittal plane, measured on a straight line
Glabelle-lambda length (GLL)	g-l	Direct distance from the glabella to lambda
Cranial base length (CBL)	ba-n	Direct distance from the basion to nasion
Basion-bregma height (BBH)	ba-b	Direct distance from the basion to bregma
Basion-nasospinale length (BNSL)	ba-ns	Direct distance from the basion to nasospinale
Foramen magnum length (FOL)	ba-o	Direct distance from the basion to opisthion
Frontal chord (FRC)	n-b	Direct distance from the nasion to bregma taken in the midsagittal plane
Nasio-occipital length (NOL)	n-op	Distance between the nasion and opisthocranion in the midsagittal plane, measured on a straight line
Nasal height (NLH)	n-ns	Direct distance from the nasion to the midpoint of a line connecting the lowest points of the inferior margin of the nasal notches
